# High *RAB25* expression is associated with good clinical outcome in patients with locally advanced head and neck squamous cell carcinoma

**DOI:** 10.1002/cam4.153

**Published:** 2013-10-31

**Authors:** Marta Téllez-Gabriel, Irene Arroyo-Solera, Xavier León, Alberto Gallardo, Montserrat López, Maria V Céspedes, Isolda Casanova, Antonio López-Pousa, Miquel Quer, Maria A Mangues, Agustí Barnadas, Ramón Mangues, Miguel A Pavón

**Affiliations:** 1 Grup d'Oncogènesi i Antitumorals (GOA), Institut d'Investigacions Biomèdiques Sant Pau (IIB-Sant Pau)Barcelona, Spain; 2 Centro de Investigación Biomédica en Red en Bioingeniería, Biomateriales y Nanomedicina (CIBER-BBN)Barcelona, Spain; 3 Department of Otorhinolaryngology (ORL), Hospital de la Santa Creu i Sant PauBarcelona, Spain; 4 Department of Pathology, Clínica GironaGirona, Spain; 5 Department of Medical Oncology, Hospital de la Santa Creu i Sant PauBarcelona, Spain; 6 Department of Pharmacy, IIB-Sant Pau, Hospital de la Santa Creu i Sant PauBarcelona, Spain

**Keywords:** HNSCC, prognosis, RAB25, tumor suppressor

## Abstract

Currently there are no molecular markers able to predict clinical outcome in locally advanced head and neck squamous cell carcinoma (HNSCC). In a previous microarray study, *RAB25* was identified as a potential prognostic marker. The aim of this study was to analyze the association between *RAB25* expression and clinical outcome in patients with locally advanced HNSCC treated with standard therapy. In a retrospective immunohistochemical study (*n* = 97), we observed that *RAB25*-negative tumors had lower survival (log-rank, *P* = 0.01) than patients bearing positive tumors. In an independent prospective mRNA study (*n* = 117), low *RAB25* mRNA expression was associated with poor prognosis. Using classification and regression tree analysis (CART) we established two groups of patients according to their *RAB25* mRNA level and their risk of death. Low mRNA level was associated with poor local recurrence-free (log-rank, *P* = 0.005), progression-free (log-rank, *P* = 0.002) and cancer-specific (log-rank, *P* < 0.001) survival. Multivariate Cox model analysis showed that low expression of *RAB25* was an independent poor prognostic factor for survival (hazard ratio: 3.84, 95% confidence interval: 1.93–7.62, *P* < 0.001). Patients whose tumors showed high *RAB25* expression had a low probability of death after treatment. We also found lower *RAB25* expression in tumors than in normal tissue (Mann–Whitney *U*, *P* < 0.001). Moreover, overexpression of *RAB25* in the UM-SCC-74B HNSCC cell line increased cisplatin sensitivity, and reduced cell migration and invasion. Our findings support a tumor suppressor role for *RAB25* in HNSCC and its potential use to identify locally advanced patients with a high probability of survival after genotoxic treatment.

## Introduction

Head and neck squamous cell carcinoma (HNSCC) is the sixth most common cancer in developed countries [1, 2]. In recent years, chemotherapy combined with radiotherapy (RT) and/or surgery has increased organ preservation and improved locoregional control in locally advanced patients [3]. However, despite the success of these new combination strategies, the 5-year survival rate remains at approximately 50% [2, 4]. A high percentage of patients suffer relapse, developing lymph node or distant metastases, or secondary tumors [5, 6].

To date, response to induction chemotherapy (IC) and the infection by human papillomavirus (HPV) are the only markers used in locally advanced HNSCC treatment decisions [7–9]. Nevertheless, IC response is not always effective in predicting response to subsequent RT or chemoradiotherapy (CRT). Moreover, response to IC could only be measured after three cycles of genotoxic treatment, delaying and complicating the alternative surgical resection in nonresponder patients. Regarding HPV, its prognostic and predictive value is limited to oropharyngeal HNSCC and also because of the wide variation in the incidence of HPV infection, depending on the geographic region [10]. The development of new molecular markers associated with clinical outcome in HNSCC could be useful in selecting the most appropriate treatment for each patient and to improve their follow-up.

In a previous microarray study in HNSCC patients, we identified three tumor subtypes associated with local recurrence and patient survival [11]. The poor prognosis subtype showed altered expression in genes involved in the secretory pathway and membrane trafficking. This tumor subtype underexpressed *RAB25*, a small GTPase protein that plays a role in the recycling of internalized membrane proteins and in the regulation of epithelial cell polarity [12–14].

Rab small GTPases family proteins are considered key regulators of intracellular membrane trafficking and their deregulation is involved in multiple diseases, including cancer [15–20]. Alterations in membrane trafficking and the secretory pathway could modify cell polarity and membrane receptor distribution [21]. These events could promote epithelial cell transformation and increase invasion and migration of tumor cells.

Published studies have shown that *RAB25* could play a role as a tumor suppressor or as an oncogene depending on the cell type in which it is expressed. Loss of *RAB25* expression has been associated with tumor initiation and poor prognosis in colon [22], esophageal [23] and estrogen receptor (ER)-negative breast cancer [18]. Conversely, *RAB25* overexpression has been correlated with decreased survival and increased aggressiveness in ovarian and bladder cancer [24, 25].

Recently, Amornphimoltham et al. [26] showed that overexpression of *RAB25* reduced cell invasion and metastasis in an “in vitro” and an “in vivo” model for oral cancer. Loss of *RAB25* expression has also been observed in advanced or metastatic HNSCC biopsies but its association with patient clinical outcome has not been studied yet.

In this study, we analyzed the association between *RAB25* expression and clinical outcome in two independent cohorts of patients with HNSCC. We assessed RAB25 expression in pretreatment HNSCC biopsies from a retrospective immunohistochemical study (*n* = 97) and a prospective mRNA study (*n* = 117) with locally advanced patients. We also analyzed *RAB25* expression in 19 normal tissues. Finally, we overexpressed *RAB25* in the UM-SCC-74B HNSCC cell line to analyze its effect on cell proliferation, migration, invasion, and cisplatin sensitivity.

## Methods

### Patient characteristics and treatment plan

All patients included in the prospective and retrospective studies had a pathologically confirmed, locally advanced (stages III, IVA, and IVB) HNSCC and were treated at the Hospital de la Santa Creu i Sant Pau (HSCSP) in Barcelona. Detailed clinical and pathological information of HNSCC patients are listed in Table 1. The retrospective study (*n* = 97) was performed using paraffin-embedded pretreatment tumor biopsies from locally advanced patients treated between 1995 and 2003. The prospective study (*n* = 117) was performed using fresh primary tumor biopsies obtained before they started treatment. A sample aliquot was used for pathological diagnosis of the malignancy and another aliquot was immersed in RNAlater (Applied Biosystems Incorp, Foster City, CA), frozen in cold isopentane, and kept in liquid nitrogen until RNA processing. Samples with a tumor cell percentage below 80% were excluded. Normal mucosa samples were obtained from areas without apparent clinical or histological alterations.

**Table 1:** Characteristics of patients included in the retrospective and the prospective study.

Variable	Retrospective study number of cases (%)	Prospective study number of cases (%)
Sex		
Men	91 (93.8)	109 (93.2)
Women	6 (6.2)	8 (6.8)
Tobacco consumption		
Non-smoker	4 (4.1)	5 (4.3)
<20 cigarette/day	5 (5.2)	9 (7.7)
>20 cigarette/day	85 (87.6)	101 (86.3)
Cigar or pipe	3 (3.1)	2 (1.7)
Alcohol		
Non-drinker	14 (14.4)	14 (12)
<100g/day	38 (39.2)	45 (38.5)
>100g/day	45 (46.4)	58 (49.6)
Age (years)		
<60	9 (9.3)	61 (52.1)
>60	88 (90.7)	56 (47.9)
Tumor site		
Oral cavity	8 (8.2)	16 (13.6)
Hypopharynx	18 (18.5)	16 (13.6)
Larynx	54 (55.7)	35 (30)
Oropharynx^1^	17 (17.5)	47 (40.2)
Other localizations		3 (2.6)
Tumor size		
T2	16 (16.5)	29 (24.8)
T3	56 (57.7)	55 (47.0)
T4	25 (25.8)	33 (28.2)
Node involvement		
N^+^	51 (52.6)	92 (78.6)
N^−^	46 (47.4)	25 (21.4)
TNM stage		
III	49 (50.5)	33 (28.2)
IV	48 (49.5)	84 (71.8)
Treatment		
IC followed by RT/CRT/surgery	97 (100)	73 (62.4)
Concomitant CRT		44 (37.6)

N^+^, node positive; N^−^, node negative; IC, induction chemotherapy; RT, radiotherapy; CRT, chemoradiotherapy.

1 In the retrospective study HPV status was available for 16 oropharyngeal tumors which were negative by PCR assay. In the prospective study, out of the 25 tumors with HPV data available, four were HPV16-positive.

HPV status was available for most of the oropharyngeal samples included in the retrospective and prospective study (Table 1). HPV was previously detected by a multiplex polymerase chain reaction (PCR) assay. The study was approved by the local Ethics Committee.

Patients included in these two studies were treated with concomitant CRT or IC followed by RT/CRT or surgery. IC consisted of cisplatin at a dose of 100 mg/m^2^ on day 1, and 5-FU at a dose of 1000 mg/m^2^ per day by continuous intravenous infusion on days 2–6, every 3 weeks, for three courses. After IC, patients whose tumors showed a complete response or a partial response above 80% received a conservative treatment (RT or CRT). Patients with stable or progressive disease after IC were treated with surgery, usually followed by RT. RT, at a total dose of 70 Gy, was administered in 35 fractions of 2 Gy each, over a 7-week period. CRT consisted of RT plus three cycles of cisplatin at the same doses.

In addition, we analyzed the *RAB25* expression in HNSCC samples using previously described microarray datasets available in GEO (GEO25099, GSE23558, and GSE26549) [27–29], Array Express (E-TABM-302) [30] and MIAME-VICE (Thurlow et al.) [31] public databases.

### Immunohistochemistry

Immunohistochemistry (IHC) was performed using paraffin-embedded pretreatment tumor biopsies. Cell block sections or 5-μm tissue samples were deparaffinized in xylol and rehydrated using decreasing ethanol concentrations (100%, 96%, 80%, 70%, and 50%). The samples were immersed in Target Retrieval Solution at pH 5.6 (DakoCytomation S.A., St Just Desvern, Spain) and autoclaved for 10 min at 121°C for antigenic retrieval. Endogenous tissue peroxidase was inactivated by immersing the samples in a 3% H_2_O_2_ solution for 10 min. Samples were then incubated with *RAB25* monoclonal antibody (clone 3F12F3; Abnova, Taipei, Taiwan) at 1:1000 dilution. For primary antibody detection, we used the Flex EnvisionKit in an automatic Dako Autostainer System (DakoCytomation S.A., St Just Desvern, Spain), according to the manufacturer's instructions. Counterstaining was performed with hematoxylin (DakoCytomation S.A., St Just Desvern, Spain). Samples were dehydrated in a growing ethanol and xylol gradient, and mounted with DPX media (Sigma Aldrich, Tres Cantos, Spain). Negative controls were processed with non-immunized mouse serum instead of the primary antibody. Immunostained sections were quantified by three independent observers. We registered the presence of *RAB25*-positive or negative cells in three 200× fields using a bright-field light microscope. Tumors were classified as positive if they had more than 1% *RAB25*-positive cells. Negative tumors included samples lacking *RAB25* staining or samples with less than 1% of positive cells.

### RNA extraction and real-time RT-PCR

Total RNA was extracted with Trizol (Invitrogen Ltd, Paisley, U.K.) as previously described and cleaned using RNeasy® Spin columns (Qiagen Incorp, Valencia, CA). Total RNA was quantified spectrophotometrically. Reverse transcription was performed using 1.5 μg of total RNA and the High Capacity cDNA Archive Kit (Life Technologies Ltd, Paisley, U.K.), in a 50 μL final reaction volume, containing 5 μL of RT buffer, 2 μL of dNTPs mixture, 5 μL of Random Hexamer Primers, 125 U of Multiscribe Reverse transcriptase, and 40 U of RNase inhibitor (Life Technologies Ltd, Paisley, U.K.). These mixtures were incubated at 25°C for 20 min, and then at 37°C for 2 h. Finally, heating at 95°C for 3 min was used to inactivate the reverse transcriptase.

mRNA expression was measured on an ABIPRISM 7000 Sequence Detection System (Life Technologies Ltd, Paisley, U.K.) using predesigned Taqman® Gene Expression Assays for *RAB25* (Hs00220628_m1) and *HPRT1* (Hs99999909_m1) (Life Technologies Ltd, Paisley, U.K.), as previously described [32]. The *HPRT1* gene was used as the endogenous control and the RNA obtained from the HNSCC UM-SCC-22A cell line was used as the calibration sample.

### Cell lines and reagents

UM-SCC-22A, UM-SCC-22B, UM-SCC-74B, FaDu, SCC9, and SCC25 HNSCC cell lines were used for in vitro assays. SCC25, SCC9 and FaDu were obtained from the ATCC (http://www.lgcstandards-atcc.org). UM-SCC-22A and UM-SCC-22B were a generous gift from Dr. R. H. Brakenhoff [33] and UM-SCC-74B by Dr. Gregory Oakley [34]. Oral normal mucosa OKF6-tert1 keratinocytes were obtained from the Cell Culture Core of the Harvard Skin Disease Research Center (http://www.rheinwaldlab.bwh.harvard.edu). UM-SCC-22A, UM-SCC-22B, UM-SCC-74B, and FaDu were grown in DMEM (Life Technologies Ltd, Paisley, U.K.) containing 10% of FBS (Life Technologies Ltd, Paisley, U.K.), 2 mmol/L glutamine and 50 U/mL penicillin/streptomycin. SCC9 and SCC25 cell lines were grown in DMEM:F12 medium (1:1) supplemented with 40 ng/mL of hydrocortisone, 2 mmol/L glutamine, and 50 U/mL penicillin/streptomycin. OKF6-tert1 was cultured in Keratinocyte Serum Free Medium (K-SFM, Life Technologies Ltd, Paisley, U.K.) supplemented with Epidermal Growth Factor, CaCl_2_, and pituitary extract. All cell lines were cultured in a humidified atmosphere at 37°C and 5% of CO_2_. Cell lines were authenticated using the Cell ID kit (Promega Corporation, Madison, WI). Obtained short tandem repeat (STR) profiles were compared with the original STR profiles previously described [35, 36].

Transfection was performed using Lipofectamine 2000 (Life Technologies, Paisley, U.K.) according to the manufacturer's protocol. Cells were seeded in a six-well plate and cotransfected with pEGF-N1 (100 ng) and pCDNA3-*RAB25* (600 ng) gift by Dr. K.-Wai Cheng [24]. Cells were selected for 2 weeks in 750 μg/mL of G418 (Life Technologies Ltd, Paisley, U.K.) and then grown as individual clones. *RAB25* overexpression in selected clones was evaluated by quantitative PCR.

### Proliferation, cytotoxicity, and apoptosis assays

Proliferation assays were performed seeding cells at a density of 30,000 cells/well in six-well/plates. We counted cells after 48, 96, and 144 h of growth, using the Cell Scepter electronic cell counter (Millipore corporation, Billerica, MA). Cell proliferation analysis was also performed using the XTT Cell proliferation kit II assay (Roche Diagnostics, Mannheim, Germany). We seeded 5000 cells/well in 96-well plates and quantified the number of viable cells after 24 h, 48 h, and 72 h. Experiments were performed three times and each experiment included five replicates for each cell line and time. Differences between parental and *RAB25* overexpressing cells were analyzed applying the Mann–Whitney *U* test.

Cytotoxicity assays were performed seeding cells in 96-well plates (2500 cells/well). Cells were exposed to cisplatin (2.5–40 μmol/L) (Sigma-Aldrich Quimica SA, Madrid, Spain) dissolved in saline buffer. We determined cell sensitivity to cisplatin after 48 h exposure using the XTT Cell proliferation kit II assay (Roche Diagnostics, Mannheim, Germany). Assays were performed at least three times in 96-well plates. Each cisplatin concentration was represented by five wells of cells.

To determine cell death induction, we treated cells with a 15 μmol/L concentration of cisplatin for 24 h and stained them with ProLong® Gold Antifade DAPI (Life Technologies Ltd, Paisley, U.K.) following the manufacturer's protocol.

### Scattering, migration, and invasion assays

For scattering assays, cells were seeded in a 100-mm-diameter plate, previously coated with Poly-Hema (Sigma-Aldrich Quimica SA, Madrid, Spain), at a density of 200,000 cells/plate to promote cell aggregates. Twelve hours later, we transferred cells to a 100-mm-diameter plate pretreated with collagen to allow for cell attachment. After 24 h, we analyzed the presence of cell scattering.

Migration and invasion assays were performed using 24-well transwell plates and inserts with 8-μm pore size (Corning, Inc., Corning, NY). Cells were previously deprived of FBS for 12 h, seeded in a migration chamber at a density of 2500 cells/well and incubated in a 24-well plate containing DMEM with 10% FBS. After 48 h, we removed cells that remained on the top side of the filter using a cotton swab. Cells that migrated and were attached to the underside of the filter were stained with crystal violet (Sigma-Aldrich Quimica SA, Madrid, Spain). For invasion assays, we followed the same procedure but the transwell membrane was previously precoated with Matrigel (BD Biosciences, Franklin Lakes, NJ).

Transwell experiments were performed three times by duplicate. We took two 100× magnified images of each transwell membrane under the same lighting and time exposure conditions. Images were quantified using the Metamoph v5.0 sofware (Universal Imaging, Downingtown, PA), applying the HSI (HUE-saturation-intensity) model as previously described [32]. Using the set color threshold tool of Metamorph software, we established a 174-255 HUE range to select and quantify the area occupied by crystall violet-stained cells. Finally, we established the percent of area occupied by the stained cells on the total image area, which was a measurement of cell migration or invasion.

### Bisulfite sequencing

Methylation analysis of Rab25 promoter focused on the CpG14 island previously described by Tong et al. [23]. Genomic DNA was isolated from UM-SCC-74B, UM-SCC-22A, UM-SCC-22B, and OKF6 Tert1 cell lines using the QIAmp DNA Mini kit (Qiagen Incorp, Valencia, CA). A quantity of 2 mcg of genomic DNA was bisulfite-converted using the Cells-to-CpG Bisulfite conversion kit (Life Technologies Ltd, Paisley, U.K.).

To amplify and sequence the CpGI4 island we used the primers previously described by Tong et al. (Forward: TGTTATTTAGGTTGGAGTGTAAGGG; reverse: CAACCAAAAAAATTAAAAACCAACTCA). Bisulfite-converted genomic DNA was amplified in a 50 μL final reaction volume, containing 1.0 U of Platinum Taq DNA Polymerase High Fidelity (Life Technologies Ltd, Paisley, U.K.), 5 μL of 10× High Fidelity PCR Buffer, 2 μL of 50 mmol/L MgSO4 and 1 μL of 10 μmol/L of primers. Thermal cycling conditions were 1 min at 94°C and 45 cycles at 94°C for 30 sec, at 55°C for 30 sec and 68°C for 1 min. PCR product purification was done using QIAquick PCR Purification kit (Qiagen Incorp, Valencia, CA) and 30 ng of these products were sequenced using BigDye Terminador v3.1 Cycle Sequencing kit (Life Technologies Ltd, Paisley, U.K.). The sequencing reaction products were analyzed on a ABI 3130xl Genetic Analyzer and using the Chromas Lite 2.1.1 software.

### 5-aza-dC treatment

UM-SCC-74B, UM-SCC-22A, and UM-SCC-22B cells were treated with 1 μmol/L-and 50-μmol/L concentrations of the DNA methyltransferase inhibitor 5-aza-dC (5-aza-2′-deoxycytidine, Sigma-Aldrich Quimica SA, Madrid, Spain) during 72 h. Total RNA was isolated using TRIzol reagent (Life Technologies Ltd, Paisley, U.K.). *RAB25* quantitative RT-PCR analysis was performed as described for tumor samples.

### Statistical data analysis

Local recurrence-free survival (LRFS) was defined as the time from diagnosis to recurrence at the primary tumor location or the latest follow-up date. Progression-free survival (PFS) was defined as the time from diagnosis to recurrence at the primary tumor location, nodal recurrence or metastasis, or the latest follow-up date. Cancer-specific survival (CSS) was defined as the interval from the date of diagnosis to the date of death from HNSCC or to the latest follow-up date. Patients were censored from the CSS analysis at the date of death if they died from other causes than cancer.

To assess the association between gene expression and LRFS, PFS or CSS we applied univariate and multivariate Cox regression model analysis. Patients were classified in two groups according to their risk of death, as a function of having low- or high-*RAB25* mRNA levels, using classification and regression tree analysis (CART) [37]. Kaplan–Meier curves and log-rank test were used to identify differences in LRFS, PFS, and CSS between groups of patients established. To analyze differences between two or more conditions we used the nonparametric Mann–Whitney *U* or Kruskal–Wallis tests, respectively. Statistical analyses were performed using the SPSS v.14.01 (IBM Corporation, Armonk, NY) and R v2.15 (http://cran.es.r-project.org) software. Differences were considered significant at *P*-values <0.05 in all the applied tests.

## Results

### Loss of RAB25 protein expression in pretreatment tumor biopsies was associated with poor survival

**Figure 1:**
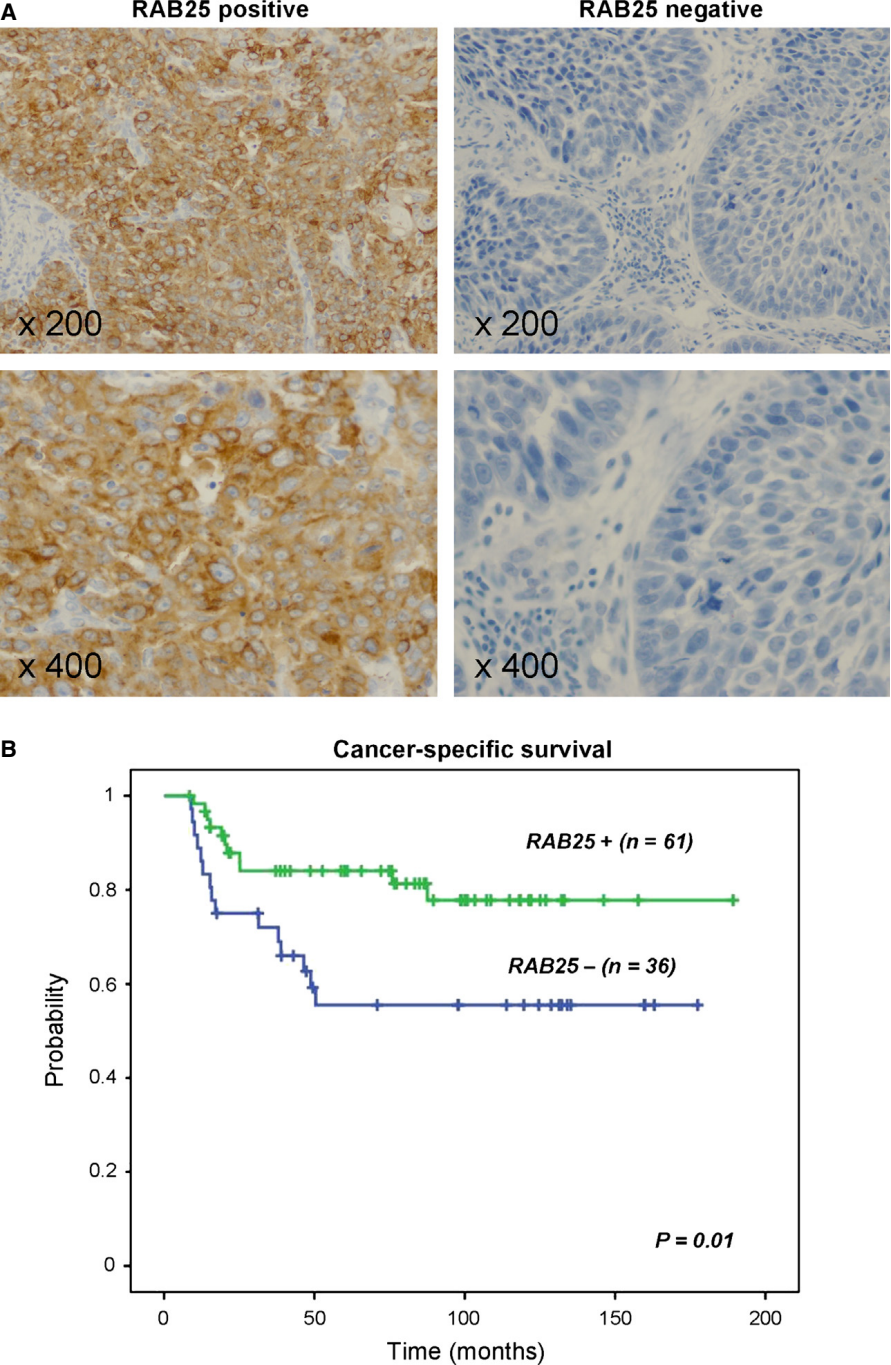
RAB25 protein expression analysis in pretreatment tumor biopsies of 97 patients included in the retrospective study. (A) Representative images of positive and negative RAB25 tumors at 200× and 400× magnifications. Images show a cytoplasmic and membrane staining pattern of RAB25. (B) Kaplan–Meier curves and log rank test showed that patients with RAB25 negative tumors have a lower cancer-specific survival (CSS) than patients bearing positive tumors.

In the retrospective study, we performed an immunohistochemical analysis of RAB25 protein expression in 97 pretreatment tumor biopsies of locally advanced HNSCC patients, who had an average follow-up time of 68 months. Patient characteristics are described in Table 1. RAB25 showed cytoplasmic and membrane staining (Fig. 1A). Normal and hyperplasic epithelial tissue adjacent to the tumor were positive for RAB25 immunostaining. Tumor stroma did not stain for *RAB25*. We observed 61 RAB25-positive tumors and 36 tumors in which RAB25 was undetectable (negative tumors).

We found significant differences in CSS based on RAB25 protein expression. Patients with RAB25 negative tumors had a shorter survival (*P* = 0.01) than patients bearing positive tumors (Fig. 1B). Multivariate Cox model analysis showed that expression of RAB25 was an independent risk factor for CSS (hazard ratio [HR]: 0.464, 95% confidence interval [CI] [0.212–1.017], *P* = 0.050) (Table S1). Node involvement was also significantly associated with patient survival (Table S1). We could not analyze the association between HPV status and CSS because all tested oropharyngeal tumors were HPV-negative.

### Low-*RAB25* mRNA expression was associated with poor local recurrence-free, progression-free, and cancer-specific survival

We confirmed the association between the expression of *RAB25* and patient survival using an independent HNSCC microarray dataset previously reported by Rickman et al. [30] (E-TABM-302). Cox model analysis showed that a high expression of *RAB25* was associated with a higher patient survival (HR: 0.64, CI 95% [0.42–0.97], *P* = 0.03).

We prospectively analyzed *RAB25* mRNA expression levels by quantitative RT-PCR in 117 pretreatment tumor biopsies obtained from patients with locally advanced HNSCC treated at the HSCSP (Table 1). The median follow-up was 29 months. The median *RAB25* mRNA expression was 3.57 (range 0.21–76.58). Cox model analysis showed a significant association between *RAB25* mRNA expression levels and survival (HR: 0.91, CI 95% [0.83–1.00], *P* = 0.05). A high expression of *RAB25* in pretreatment tumor biopsies reduced the risk of patient death.

**Figure 2:**
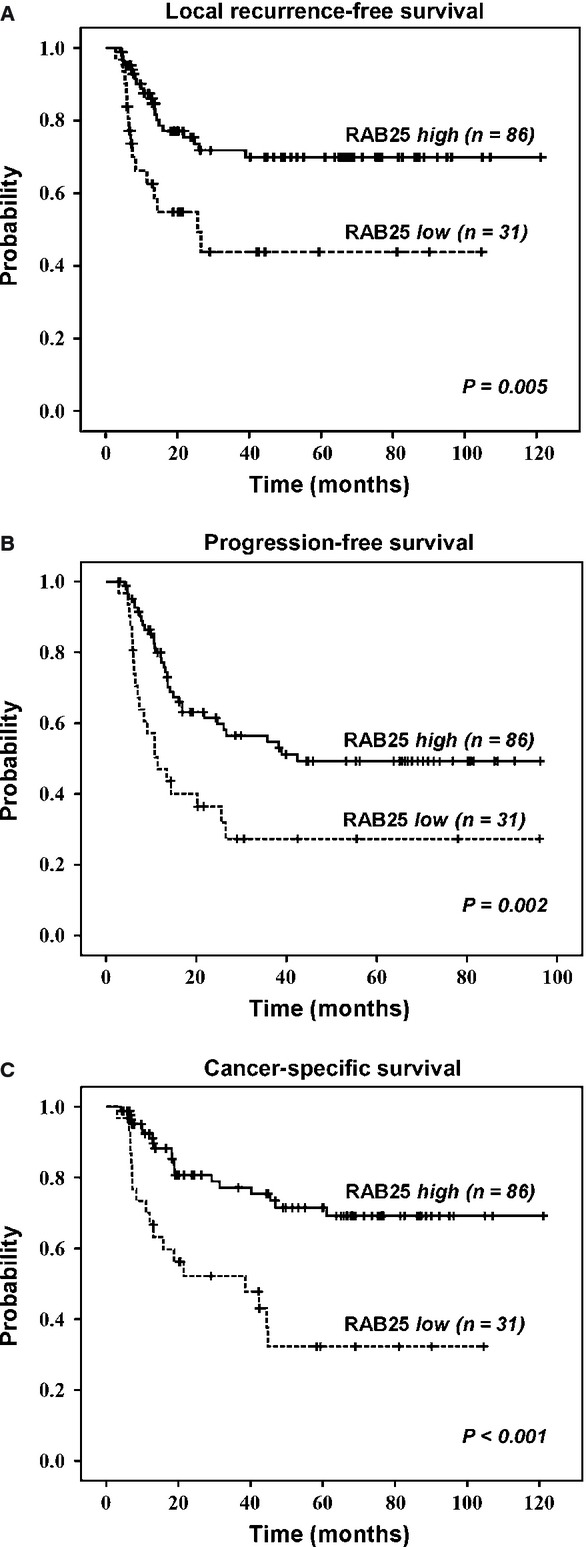
Patients included in the prospective study (*n* = 117) were stratified in two groups according to the *RAB25* mRNA level. Kaplan–Meier curves and log-rank test showed significant differences in local recurrence-free survival (A), progression-free survival (B), and cancer-specific survival (C) between patients with high expression (>1.75) and patients with low expression (<1.75).

We identified two groups of patients depending on their *RAB25*-mRNA levels in tumors (*RAB25*-mRNA level < or >1.75) using the CART. Kaplan–Meier curves showed significant differences in LRFS (*P* = 0.005), PFS (*P* = 0.002), and CSS (*P* < 0.001) between patients with *RAB25*-tumor expression above and below the established threshold (Fig. 2). The subset of patients with a low-*RAB25* expression had lower LRFS, PFS, and CSS than patients with high-*RAB25* expression. Multivariate Cox model analysis showed that *RAB25* mRNA level was an independent risk factor for LRFS (HR: 3.00, 95% CI [1.46–6.17], *P =* 0.003), PFS (HR: 2.49, 95% CI [1.41–4.41], *P* = 0.002), and CSS (HR: 3.84, 95% CI [1.93–7.62], *P* < 0.001) in locally advanced HNSCC patients (Table 2). HPV status was available for 29 of the oropharyngeal tumors included in the prospective study. Only four of these tumors were positive for HPV (HPV16). Three of these patients showed a high tumor expression of *RAB25* and were alive at the end of the study. The fourth patient, who had a low tumor expression of *RAB25*, died from a cause unrelated to the HNSCC. The low-HPV infection rate in our series of patients did not allow to study the association between HPV infection and Rab25 expression and clinical outcome.

**Table 2:** Multivariate Cox model analysis of local recurrence-free, progression-free, and cancer-specific survival in the prospective cohort (*n*=117).

	Local recurrence-free survival		Progression-free survival		Cancer-specific survival	
			
	HR (95% CI)	*P*	HR (95% CI)	*P*	HR (95% CI)	*P*
All patients						
RAB25	3.00 (1.46–6.17)	**0.003**	2.49 (1.41–4.41)	**0.002**	3.84 (1.93–7.62)	**<0.001**
Age	0.53 (0.25–1.10)	0.087	0.79 (0.45–1.40)	0.429	0.58 (0.29–1.17)	0.130
Alcohol consumption	2.05 (0.59–7.04)	0.253	1.83 (0.64–5.22)	0.258	1.33 (0.31–5.68)	0.702
Tobacco consumption	0.30 (0.03–3.08)	0.314	0.24 (0.03–2.18)	0.203	0.49 (0.04–5.66)	0.568
Sex	0.34 (0.09–1.27)	0.107	0.79 (0.23–2.70)	0.706	0.53 (0.11–2.43)	0.410
N^−^ vs. N^+^	0.67 (0.28–1.64)	0.388	0.58 (0.28–1.19)	0.136	0.61 (0.24–1.53)	0.287
Tumor size	0.52 (0.20–1.31)	0.166	0.59 (0.29–1.21)	0.151	0.76 (0.33–1.77)	0.527
Localization	1.94 (0.93–4.04)	*0.078*	1.32 (0.75–2.32)	0.334	1.86 (0.92–3.77)	0.084
CRT/IC+RT/IC+CRT						
RAB25	2.43 (1.08–5.50)	**0.033**	2.29 (1.17–4.49)	**0.016**	4.60 (1.88–11.25)	**0.001**
Age	0.43 (0.18–1.01)	0.053	0.77 (0.38–1.57)	0.472	0.74 (0.29–1.87)	0.526
Alcohol consumption	2.09 (0.67–6.56)	0.206	1.67 (0.49–5.66)	0.411	0.85 (0.11–6.53)	0.877
Tobacco consumption	0.52 (0.05–6.00)	*0.599*	0.75 (0.65–8.58)	0.813	2.46 (0.13–45.85)	0.546
Sex	0.31 (0.81–1.18)	0.086	0.65 (0.19–2.29)	0.507	0.39 (0.08–1.93)	0.250
N^−^ vs. N^+^	0.61 (0.23–1.63)	0.324	0.60 (0.26–1.39)	0.236	0.69 (0.22–2.18)	0.528
Tumor size	0.29 (0.09–0.89)	**0.031**	0.44 (0.18–1.08)	0.074	0.64 (0.21–1.92)	0.424
Localization	1.48 (0.64–3.42)	0.359	1.48 (0.74–2.95)	0.271	2.87 (1.08–7.61)	**0.034**

HR, hazard ratio; 95% CI, 95% confidence interval; RAB25, RAB25 mRNA level <1,7 versus RAB25 mRNA level >1,7; N^−^ versus N^+^, node negative versus node positive; Tumor size, T1–T2 versus T3–T4; Localization, oropharynx or oral cavity versus hypopharynx or larynx.

Statistically significant P-values are indicated in bold (*P*-value < 0.05).

We next searched for possible differences in LRFS, PFS, and CSS within the subset of patients who received CRT/RT after IC or concomitant CRT (*n* = 87), excluding those treated with surgery after IC (*n* = 23). Again, multivariate Cox model confirmed that *RAB25* mRNA level was an independent risk factor for PFS and CSS (Table 2). We used CART analysis to generate a model to predict death in patients who had followed a genotoxic treatment (Fig. S1). Again, CART analysis selected the 1.75 mRNA level as the best cut-off to classify patients in two risk groups: a high-risk group whose tumors expressed a *RAB25* mRNA level below 1.75 and a low-risk group with a *RAB25*-tumor mRNA level above 1.75. Specificity and sensitivity in predicting patient death were 81.3% and 65.5%, respectively.

### HNSCC tumors express lower *RAB25* levels than normal mucosa

**Figure 3:**
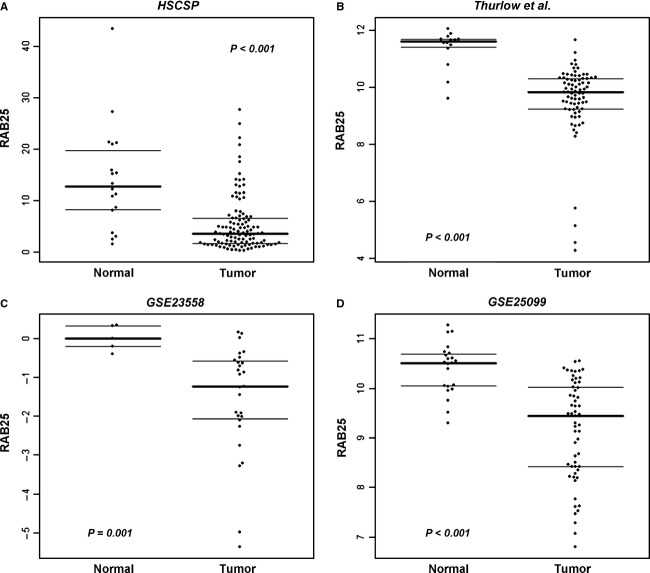
(A) Box plots showed a lower *RAB25* expression in tumor tissue (*n* = 117) than in normal tissue (*n* = 19). We confirmed significant differences using three independent microarrays datasets (B) Thurlow et al. (C) GSE23558 and (D) GSE25099 (Mann–Whitney *U* test).

We compared *RAB25* expression levels between 19 normal mucosa and 117 tumor samples. Tumor tissue had a lower expression of *RAB25* than normal tissue (Fig. 3A). We confirmed these results by analyzing *RAB25* expression in three previously reported microarray datasets [27, 28, 31]. We found significant differences in *RAB25* mRNA expression levels between tumor and mucosa tissues in all the analyzed datasets. Tumor tissue showed a lower expression of *RAB25* than normal tissue (Fig. 3B–D). Analysis using the dataset described by Saintigny et al. [29] (GSE26549) showed no significant differences in *RAB25*-mRNA levels of precursor HNSCC lesions with different histological grades (hyperplasia vs. dysplasia) (Fig. S2).

### *RAB25* overexpression increases cell proliferation and sensitivity to cisplatin whereas it reduces cell invasion and migration

**Figure 4:**
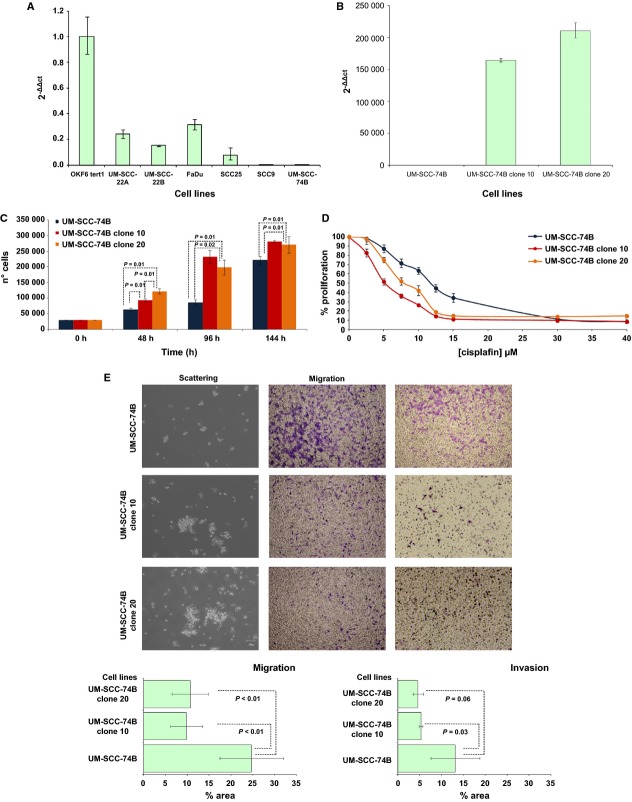
(A) *RAB25* mRNA levels analyzed in HNSCC cell lines by quantitative RT-PCR. (B) UM-SCC-74B clones overexpressing *RAB25* detected by qRT-PCR. (C) Proliferation assays performed seeding cells at a density of 30,000 cells/well in 6 well/plates, during 48, 96, and 144 h. (D) XTT cytoxicity assays. Cells were exposed during 48 h to cisplatin (2.5–40 μmol/L). (E) Scattering, migration, and invasion assays. (Mann–Whitney *U* test). HNSCC, head and neck squamous cell carcinoma; RT-PCR, real-time polymerase chain reaction.

We analyzed *RAB25* mRNA levels in six HNSCC cell lines by quantitative RT-PCR (UM-SCC-22A, UM-SCC-22B, UM-SCC-74B, FaDu, SCC9, and SCC25) and in OKF6-Tert1 immortalized normal keratinocytes. All analyzed cell lines expressed lower *RAB25* mRNA levels than OKF6-Tert1 keratinocytes (Fig. 4A). SCC9 and UM-SCC-74B did not express detectable levels of *RAB25*. We transfected the UM-SCC-74B cell line with the pCDNA3.1-HA-*RAB25* plasmid and obtained stable clones overexpressing *RAB25* (UM-SCC-74Bclone10 and UM-SCC-74Bclone20) (Fig. 4B).

Cells overexpressing *RAB25* (UM-SCC-74B clones 10 and 20) showed a higher proliferation rate than the parental UM-SCC-74B cell line. These differences were observed at 48, 96, and 144 h after seeding (Fig. 4C). We confirmed these results using an independent technique (XTT assay). After 72 h of cell growth, UM-SCC-74B cells overexpressing *RAB25* showed a higher proliferation rate than parental UM-SCC-74B (Fig. S3A). We did not observe differences in proliferation rate between the two analyzed clones.

Cytotoxicity assays showed that *RAB25* overexpression increased cisplatin sensitivity as compared to the parental cell line (Fig. 4D). Cisplatin IC_50_ values for both clones were 50% lower than in non-*RAB25*-expressing cells. After 24 h of cisplatin exposure, levels of nuclear condensation and nuclear fragmentation in cells overexpressing *RAB25* were higher than in parental cells (Fig. S3B). However, we did not find differences in Caspase-3 activation between UM-SCC-74B and transfected cells by Western Blot analysis (data not shown).

Scattering assays showed that *RAB25* overexpression in UM*-SCC-74B* clones 10 and 20 impaired the ability to spread in coated collagen plates and promoted cell aggregation (Fig. 4E). Moreover, transwell assays showed that *RAB25* overexpression reduced cell migration and invasion rates. A reduced number of cells from UM-SCC-74B clones 10 and 20 migrated through the Matrigel coated or non-coated transwell membranes compared to the parental cell line (Fig. 4E).

Finally, we analyzed the methylation of the RAB25 promoter. Bisulfite sequencing analysis showed that methylation status of the CpGI4 island was higher in the UM-SCC-22B and UM-SCC-74B cell lines than in the UM-SCC-22A (Fig. S4). After exposure to 5-aza-20-deoxycitidine (5-aza-dC), mRNA levels of *RAB25* increased in UM-SCC-22B and UM-SCC-74B cell lines.

## Discussion

### Loss of *RAB25* expression is associated with poor survival in patients with locally advanced head and neck carcinoma

Our findings show that *RAB25* expression is associated with clinical outcome in patients with locally advanced head and neck carcinoma. Patients with tumors expressing a low level of *RAB25* had a higher risk of tumor recurrence, node or distant metastases, and death. Its role as a prognostic marker had been studied in other tumor types, but this is the first time that it is shown in patients with locally advanced HNSCC.

Studies published to date show that the role of *RAB25* in tumorigenesis and patient prognosis depends on the tumor type and the cell of origin in which *RAB25* is expressed. In agreement with colon and esophageal cancer studies [22, 23], our results in HNSCC showed that low *RAB25* expression was associated with poor survival. Similarly, in pancreatic tumors lacking CLIC3 expression, Dozynkiewicz et al. [38] also showed that low *RAB25* expression was associated with decreased survival.

Breast, bladder, and ovarian cancer studies have suggested that a high expression of *RAB25* is associated with poor prognosis [24, 25, 39]; however, these results are controversial [40, 41]. In hormone receptor-positive breast cancer, *RAB25* expression is a poor prognosis factor, whereas the breast cancer subtype with the worst prognosis, triple negative for hormonal receptors presents loss of *RAB25* expression [18, 42]. Gonzalez-Angulo et al. [41] showed that a high expression of *RAB25* was associated with higher recurrence-free survival in breast cancer patients, but they observed that *RAB25* loss its predictive value when they analyzed hormone receptor-positive tumors only. These results support the notion that the prognostic value of *RAB25* depends on the cancer cell type analyzed.

The increased sensitivity to cisplatin and cell proliferation we observed in the UM-SCC-74B cell line overexpressing *RAB25* could explain why patients with high-*RAB25* tumor expression obtain greater clinical benefit from genotoxic treatment than patients with low *RAB25* tumor expression. Cisplatin displays its maximal antitumor activity when cells are growing at the exponential phase. The increased proliferation rate observed in cells overexpressing *RAB25* could thus enhance the antitumor effect of cisplatin. After 24 h of cisplatin exposure, we found an increase in nuclear fragmentation in cells overexpressing *RAB25* but we did not find differences in Caspase-3 activation.

Regarding cell line proliferation analysis, we showed that *RAB25* expression increased cell proliferation, whereas Amornphimolthan et al. [26] found no impact on cell proliferation. A possible explanation for this discordance could be the use of different cell lines. Further research on the mechanism involved in programmed cell death induction is required to improve the understanding of the role of *RAB25* in this process.

Amornphimolthan et al. [26] study has previously shown in HNSCC that RAB25 plays an important role in tumor migration and metastasis. They showed that its re-expression in HNSCC blocked cell migration. We confirmed these results overexpressing RAB25 in the UM-SCC-74B. Loss of *RAB25* expression could therefore increase the invasion and dissemination ability of tumor cells in patients, and consequently increase the risk of recurrences and development of lymph node or distant metastases. Other authors have reported similar results in different tumor types. Tong et al. [23] showed that *RAB25* overexpression reduces the migration and invasion capacity in esophageal squamous carcinoma cell lines. Goldenring et al. showed that *RAB25* could enhance transformation and tumor invasion using the Smad3−/− mouse model of colon cancer. *RAB25*−/− Smad3+/− mice showed large invasive lesions in a high percentage of animals [22]. In contrast, *RAB25*+/+ Smad 3+/− mice did not develop adenocarcinomas. *RAB25* was also underexpressed in metastatic as compared to non-metastatic pancreatic endocrine neoplasms [43].

The most remarkable novelty of this study is that it shows the prognostic value of *RAB25* expression in patients with locally advanced HNSCC. Based on these results, determination of *RAB25* expression levels in pretreatment biopsies could identify patients with a high probability of survival. These patients could receive concomitant CRT as a first-line therapy, eliminating unnecessary surgery, and therefore increasing, organ preservation. Patients with a high probability of death, following a genotoxic treatment, could be candidates to surgery or an alternative treatment, avoiding the side effects of an ineffective genotoxic treatment.

Although we analyzed *RAB25* expression both retrospectively and prospectively, independent studies are needed to validate the sensitivity and specificity of this marker to predict treatment response. We also confirmed the prognostic value of *RAB25* using an independent microarray dataset performed with surgical patients at different tumor stages, previously published by Rickman et al. A future independent study in patients receiving genotoxic therapy could allow us to validate the predictive value of *RAB25*.

The accuracy of *RAB25* mRNA levels in distinguishing patients with a low or high risk of death after treatment was around 75%. We may improve the sensitivity and specificity of pretreatment tumor classification adding additional molecular markers, such as HPV status, which associate with HNSCC clinical outcome. In this study, we could not determine the effect of HPV on clinical outcome, due to the low incidence of HPV-positive tumors. Finally, it would be necessary to conduct a randomized trial to determine if the use of this marker for therapeutic decision making may increase disease-free survival in locally advanced HNSCC patients.

### *RAB25* as a tumor suppressor gene in HNSCC

Our findings showed that loss of *RAB25* expression is a common event in HNSCCs and supported its role as a tumor suppressor. These results are consistent with those obtained by Amornphimoltham et al. [26], who showed in a cohort of 69 patients with HNSCC, that *RAB25* was downregulated in tumor samples. Nevertheless, these authors did not study *RAB25* prognostic capacity.

Previous studies on *RAB25* have revealed that it could play a role as a tumor suppressor gene or as an oncogene depending on the cell type in which it is expressed. It has recently been suggested that *RAB25* may act as a tumor suppressor in colon, esophageal, and triple-negative breast cancers [22, 23, 44]. Goldenring et al. described that loss of *RAB25* promotes the development of intestinal neoplasia in mice [22]. Moreover, they showed that human colorectal adenocarcinomas exhibited a reduction in *RAB25* expression independently of tumor stage. Recently, Tong et al. [23] showed that esophageal squamous cell carcinoma cells (ESCC) overexpressing *RAB25* have a lower rate of tumor formation than ESCC cells with repressed *RAB25* in an in vivo model. They showed that loss of *RAB25* was associated with promoter hypermethylation on CpG14 island rather than with a chromosomal alteration. We showed that CpG14 island methylation was higher in the UM-SCC-22B and UM-SCC-74B cells. After demethylation by exposure to 5-aza-20-deoxycitidine (5-aza-dC), mRNA levels of RAB25 increased in both cell lines, suggesting that promoter hypermethylation may play a role in silencing RAB25 expression in HNSCC.

Moreover, the last two studies showed that tumor samples underexpressed *RAB25* as compared to normal tissue. These findings support the role of *RAB25* as a tumor suppressor in these tumor types. In breast and ovarian cancers, *RAB25* may have a role as a tumor suppressor or an oncogene depending on the tumor subtype in which it is expressed. *RAB25* is highly expressed in epithelial ovarian cancers but not in stromal tumors [40, 45]. Similarly, hormone receptor-positive breast tumors have a high expression of *RAB25*, whereas triple-negative breast tumors lack *RAB25* expression [18, 42]. Interestingly, the loss of expression of *RAB25* is associated with subtypes of breast or ovarian tumors displaying a mesenchymal phenotype. These findings are in agreement with our previously published microarray study in which we identified a poor prognosis subtype of HNSCC that expressed low levels of *RAB25* and showed features of epithelial–mesenchymal transition and undifferentiation [11].

Similar to findings reported in ESCC and colon cancer [22, 23, 46], our results indicate that *RAB25* could be a tumor suppressor gene in HNSCCs. We observed a loss of *RAB25* tumor expression in all four sets of samples analyzed. These results suggest that *RAB25* could play a role in epithelial transformation and tumor progression. Moreover, an analysis of the data published by Saintigny et al. [29] showed lack of differences in *RAB25* expression among precursor lesions at different histological grade, suggesting that loss of *RAB25* expression could occur during the late stages of HNSCC pathogenesis. Future studies of the molecular signaling pathways involved in proliferation, migration and apoptosis at different stages of HNSCC progression could help to clarify the role of *RAB25* in epithelial transformation and tumorigenesis.

In summary, we have shown loss of *RAB25* expression in tumor samples, an association between low *RAB25* expression and poor clinical outcome, and a reduction in cell migration and increased sensitivity to cisplatin sensitivity in *RAB25* overexpressing cells. All these findings support the role of *RAB25* as a tumor suppressor gene in HNSCC and show its value as a potential biomarker to identify locally advanced patients who are most likely to benefit from genotoxic treatment.

## Supporting Information

**Figure S1.** (A) Classification and regression tree analysis separated patients treated with genotoxic treatment in two risk groups using *RAB25* mRNA level in pretreatment tumor biopsy (<1.75 or >1.75). (B) Patients with high tumor expression of *RAB25* had a reduced risk of death than patients with low expression level.

**Figure S2.** *RAB25* mRNA level in samples with hyperplasia or dysplasia included in a public microarray dataset previously published by Saintingny et al. (GSE26549) (Mann–Whitney *U* test).

**Figure S3.** (A) Differences in cell proliferation between the parental UM-SCC-74B cell line and UM-SCC74B clones overexpressing *RAB25* using XTT assay (Mann–Whitney *U* test). (B) Hoechst assay showed a higher cell death rate in UM-SCC-74B *RAB25* overexpressing cells than in UM-SCC-74B wild-type cells, after 24 h of exposure to cisplatin (15 μmol/L).

**Figure S4.** (A) Relative methylation status of the CpGI4 island obtained by bisulfite sequencing analysis in HNSCC cell lines. *RAB25* mRNA levels measured after 72 h of treatment with 5-aza-dC of the UM-SCC-74B (B), UM-SCC-22B (C) and UM-SCC-22A (D) cell lines.

**Table S1.** Multivariate Cox model analysis of cancer-specific survival in the retrospective cohort (*n* = 97).

**Table S2.** Authentication of the HNSCC cell lines used in the study.

## References

[1] Jemal A, Bray F, Center MM, Ferlay J, Ward E, Forman D (2011). Global cancer statistics. CA Cancer J. Clin.

[2] Leemans CR, Braakhuis BJ, Brakenhoff RH (2011). The molecular biology of head and neck cancer. Nat. Rev. Cancer.

[3] Argiris A, Karamouzis MV, Raben D, Ferris RL (2008). Head and neck cancer. Lancet.

[4] Sahu N, Grandis JR (2011). New advances in molecular approaches to head and neck squamous cell carcinoma. Anticancer Drugs.

[5] Pfister DG, Ang KK, Brizel DM, Burtness BA, Cmelak AJ, Colevas AD (2011). Head and neck cancers. J. Natl. Compr. Canc. Netw.

[6] Leon X, Martinez V, Lopez M, Garcia J, Quer M (2010). Risk of third and fourth tumors in patients with head and neck cancer. Head Neck.

[7] Sethi S, Ali-Fehmi R, Franceschi S, Struijk L, Quint LJ, van Doorn W (2012). Characteristics and survival of head and neck cancer by HPV status: a cancer registry-based study. Int. J. Cancer.

[8] Ragin CC, Taioli E (2007). Survival of squamous cell carcinoma of the head and neck in relation to human papillomavirus infection: review and meta-analysis. Int. J. Cancer.

[9] Argiris A (2005). Induction chemotherapy for head and neck cancer: will history repeat itself?. J. Natl. Compr. Canc. Netw.

[10] Marklund L, Nasman A, Ramqvist T, Dalianis T, Munck-Wikland E, Hammarstedt L (2012). Prevalence of human papillomavirus and survival in oropharyngeal cancer other than tonsil or base of tongue cancer. Cancer Med.

[11] Pavon M, Parreno M, Tellez-Gabriel M, Sancho F, Lopez M, Cespedes M (2012). Gene expression signatures and molecular markers associated with clinical outcome in locally advanced head and neck carcinoma. Carcinogenesis.

[12] Tang BL (2010). Is Rab25 a tumor promoter or suppressor – context dependency on RCP status?. Tumour Biol.

[13] Goldenring JR, Nam KT (2011). Rab25 as a tumour suppressor in colon carcinogenesis. Br. J. Cancer.

[14] Krishnan M, Lapierre LA, Knowles BC, Goldenring JR (2013). Rab25 regulates integrin expression in polarized colonic epithelial cells. Mol. Biol. Cell.

[15] Stenmark H (2009). Rab GTPases as coordinators of vesicle traffic. Nat. Rev. Mol. Cell Biol.

[16] Pylypenko O, Goud B (2012). Posttranslational modifications of Rab GTPases help their insertion into membranes. Proc. Natl. Acad. Sci. USA.

[17] Grosshans BL, Ortiz D, Novick P (2006). Rabs and their effectors: achieving specificity in membrane traffic. Proc. Natl. Acad. Sci. USA.

[18] Agarwal R, Jurisica I, Mills GB, Cheng KW (2009). The emerging role of the RAB25 small GTPase in cancer. Traffic.

[19] Ho JR, Chapeaublanc E, Kirkwood L, Nicolle R, Benhamou S, Lebret T (2012). Deregulation of Rab and Rab effector genes in bladder cancer. PLoS One.

[20] Kelly EE, Horgan CP, Goud B, McCaffrey MW (2012). The Rab family of proteins: 25 years on. Biochem. Soc. Trans.

[21] Tanos B, Rodriguez-Boulan E (2008). The epithelial polarity program: machineries involved and their hijacking by cancer. Oncogene.

[22] Nam KT, Lee HJ, Smith JJ, Lapierre LA, Kamath VP, Chen X (2010). Loss of Rab25 promotes the development of intestinal neoplasia in mice and is associated with human colorectal adenocarcinomas. J. Clin. Invest.

[23] Tong M, Chan KW, Bao JY, Wong KY, Chen JN, Kwan PS (2012). Rab25 is a tumor suppressor gene with anti-angiogenic and anti-invasive activities in esophageal squamous cell carcinoma. Cancer Res.

[24] Cheng KW, Lahad JP, Kuo WL, Lapuk A, Yamada K, Auersperg N (2004). The RAB25 small GTPase determines aggressiveness of ovarian and breast cancers. Nat. Med.

[25] Zhang J, Wei J, Lu J, Tong Z, Liao B, Yu B (2013). Overexpression of Rab25 contributes to metastasis of bladder cancer through induction of epithelial-mesenchymal transition and activation of Akt/GSK-3beta/Snail signaling. Carcinogenesis.

[26] Amornphimoltham P, Rechache K, Thompson J, Masedunskas A, Leelahavanichkul K, Patel V (2013). Rab25 regulates invasion and metastasis in head and neck cancer. Clin. Cancer Res.

[27] Peng CH, Liao CT, Peng SC, Chen YJ, Cheng AJ, Juang JL (2011). A novel molecular signature identified by systems genetics approach predicts prognosis in oral squamous cell carcinoma. PLoS One.

[28] Ambatipudi S, Gerstung M, Pandey M, Samant T, Patil A, Kane S (2012). Genome-wide expression and copy number analysis identifies driver genes in gingivobuccal cancers. Genes Chromosom. Cancer.

[29] Saintigny P, Zhang L, Fan YH, El-Naggar AK, Papadimitrakopoulou VA, Feng L (2011). Gene expression profiling predicts the development of oral cancer. Cancer Prev. Res. (Phila.).

[30] Rickman DS, Millon R, Thomas A, De Reynies E, Wasylyk C, Muller D (2008). Prediction of future metastasis and molecular characterization of head and neck squamous-cell carcinoma based on transcriptome and genome analysis by microarrays. Oncogene.

[31] Thurlow JK, Pena Murillo CL, Hunter KD, Buffa FM, Patiar S, Betts G (2010). Spectral clustering of microarray data elucidates the roles of microenvironment remodeling and immune responses in survival of head and neck squamous cell carcinoma. J. Clin. Oncol.

[32] Pavon MA, Parreno M, Leon X, Sancho FJ, Cespedes MV, Casanova I (2008). Ku70 predicts response and primary tumor recurrence after therapy in locally advanced head and neck cancer. Int. J. Cancer.

[33] Klaassen I, Brakenhoff RH, Smeets SJ, Snow GB, Braakhuis BJ (2001). Expression of retinoic acid receptor gamma correlates with retinoic acid sensitivity and metabolism in head and neck squamous cell carcinoma cell lines. Int. J. Cancer.

[34] Manthey KC, Glanzer JG, Dimitrova DD, Oakley GG (2010). Hyperphosphorylation of replication protein A in cisplatin-resistant and -sensitive head and neck squamous cell carcinoma cell lines. Head Neck.

[35] Brenner JC, Graham MP, Kumar B, Saunders LM, Kupfer R, Lyons RH (2010). Genotyping of 73 UM-SCC head and neck squamous cell carcinoma cell lines. Head Neck.

[36] Zhao M, Sano D, Pickering CR, Jasser SA, Henderson YC, Clayman GL (2011). Assembly and initial characterization of a panel of 85 genomically validated cell lines from diverse head and neck tumor sites. Clin. Cancer Res.

[37] Aviles-Jurado FX, Leon X (2013). Prognostic factors in head and neck squamous cell carcinoma: comparison of CHAID decision trees technology and cox analysis. Head Neck.

[38] Dozynkiewicz MA, Jamieson NB, Macpherson I, Grindlay J, van den Berghe PV, von Thun A (2012). Rab25 and CLIC3 collaborate to promote integrin recycling from late endosomes/lysosomes and drive cancer progression. Dev. Cell.

[39] Yin YX, Shen F, Pei H, Ding Y, Zhao H, Zhao M (2012). Increased expression of Rab25 in breast cancer correlates with lymphatic metastasis. Tumour Biol.

[40] Sheach LA, Adeney EM, Kucukmetin A, Wilkinson SJ, Fisher AD, Elattar A (2009). Androgen-related expression of G-proteins in ovarian cancer. Br. J. Cancer.

[41] Gonzalez-Angulo AM, Liu S, Chen H, Chavez-Macgregor M, Sahin A, Hortobagyi GN (2013). Functional proteomics characterization of residual breast cancer after neoadjuvant systemic chemotherapy. Ann. Oncol.

[42] Cheng JM, Ding M, Aribi A, Shah P, Rao K (2006). Loss of RAB25 expression in breast cancer. Int. J. Cancer.

[43] Hansel DE, Rahman A, House M, Ashfaq R, Berg K, Yeo CJ (2004). Met proto-oncogene and insulin-like growth factor binding protein 3 overexpression correlates with metastatic ability in well-differentiated pancreatic endocrine neoplasms. Clin. Cancer Res.

[44] Cheng JM, Volk L, Janaki DK, Vyakaranam S, Ran S, Rao KA (2009). Tumor suppressor function of Rab25 in triple-negative breast cancer. Int. J. Cancer.

[45] Zhao M, Yin YX, Guo F, Yang YY, Shen F, Chen Q (2012). Increased Rab25 expression is not correlated with peritoneal metastasis of ovarian cancers. Cancer Invest.

[46] Luthra MG, Ajani JA, Izzo J, Ensor J, Wu TT, Rashid A (2007). Decreased expression of gene cluster at chromosome 1q21 defines molecular subgroups of chemoradiotherapy response in esophageal cancers. Clin. Cancer Res.

