# Supplementary figures and images for: High RAB25 expression is associated with good clinical outcome in patients with locally advanced head and neck squamous cell carcinoma
Source: Cancer Med. 2013 Oct 31;2(6):950–63. doi: 10.1002/cam4.153 (PMC3892400; doi:10.1002/cam4.153)

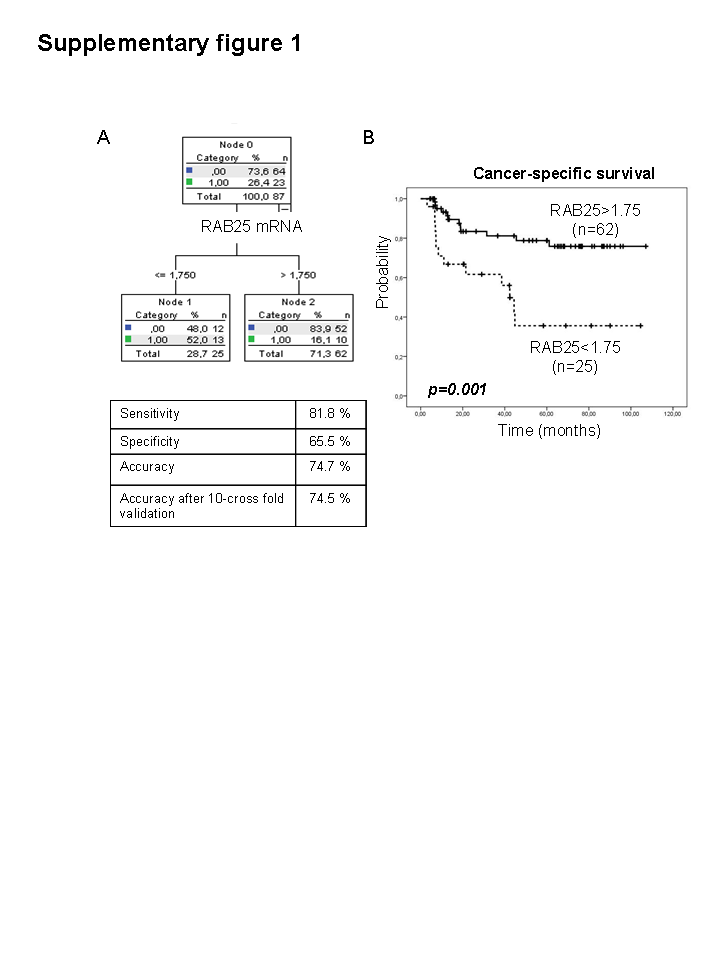

Supplement: Supplementary file 1 [file cam40002-0950-SD1.tif]

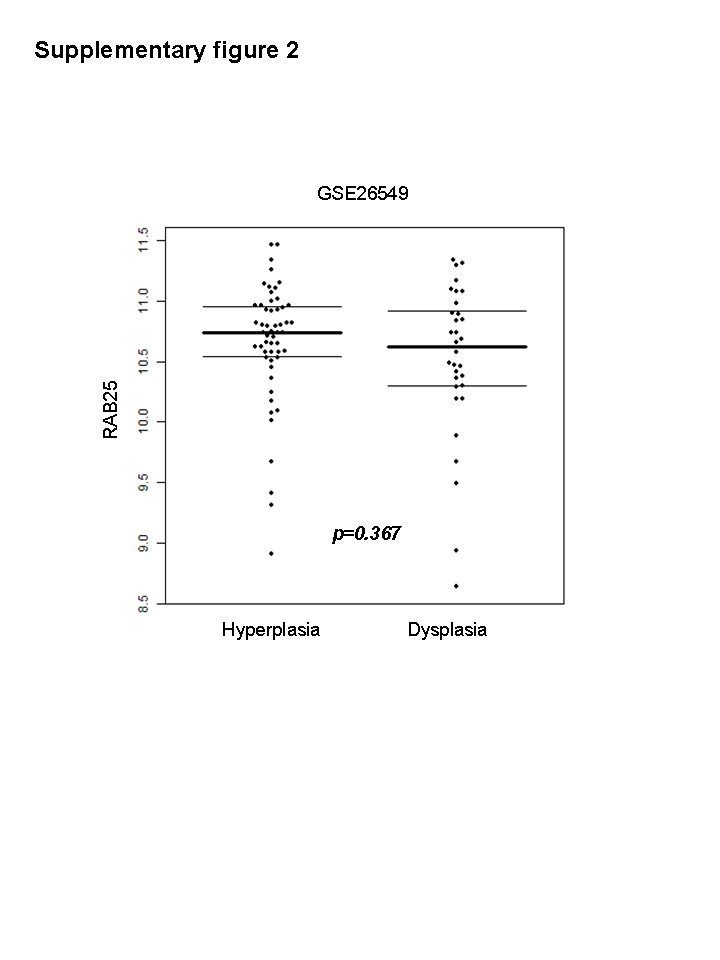

Supplement: Supplementary file 2 [file cam40002-0950-SD2.tif]

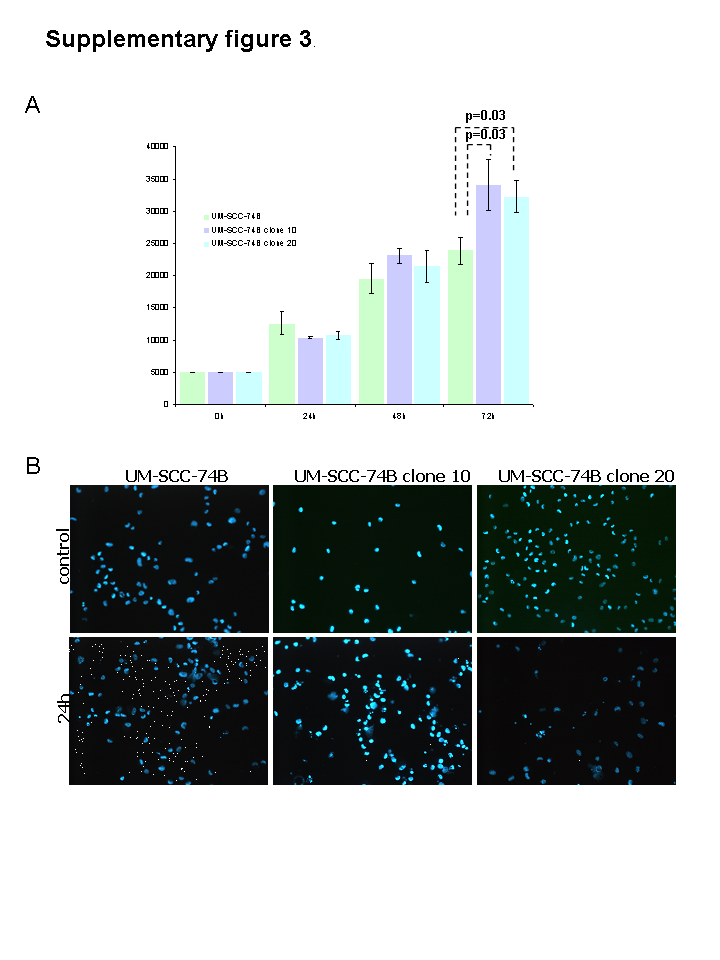

Supplement: Supplementary file 3 [file cam40002-0950-SD3.tif]

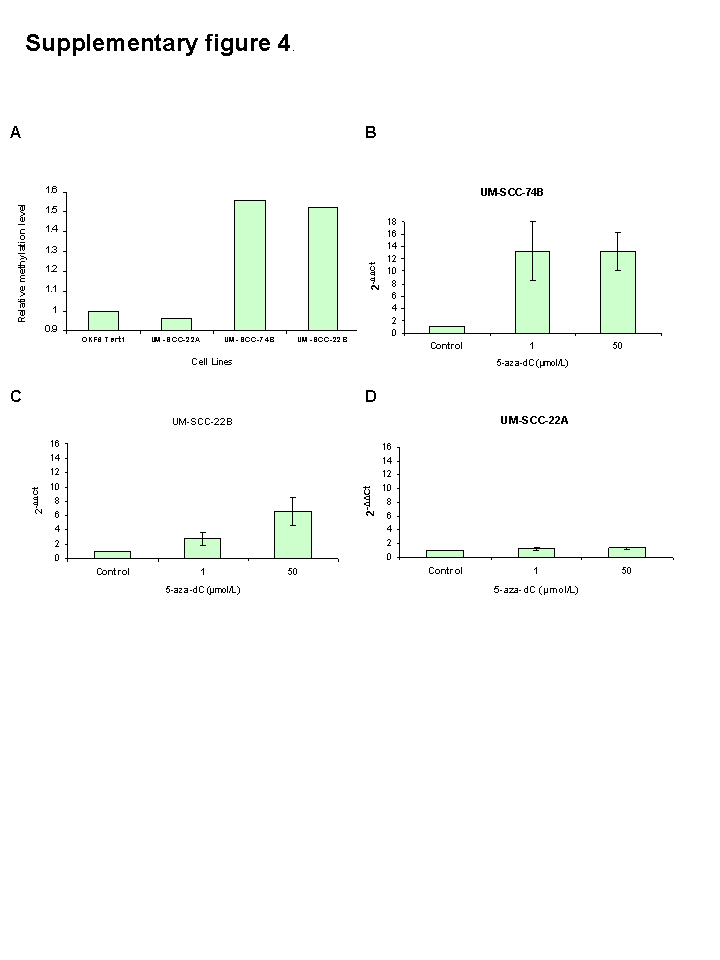

Supplement: Supplementary file 4 [file cam40002-0950-SD4.tif]
