# Supplementary material for: High RAB25 expression is associated with good clinical outcome in patients with locally advanced head and neck squamous cell carcinoma
Source: Cancer Med. 2013 Oct 31;2(6):950–63. doi: 10.1002/cam4.153 (PMC3892400; doi:10.1002/cam4.153)
Supplement: Supplementary file 5 [file cam40002-0950-SD5.doc]

Supplementary table 1. Multivariate Cox model analysis of cancer-specific survival in the retrospective cohort (n=97).

|  | **Multivariate** | |
| --- | --- | --- |
| RAB25 IHQ^c^  Sex^d^  Age^e^  Alcohol consumption  Tobacco consumption  N^+^ vs N ^- h^  Tumor size ^i^ | **HR^a^ (95% CI^b^)**  0.464 (0.212-1.017)  2.705 (0.810-9.039)  0.537 (0.185-1.560)  1.931 (0.876-4.259)  0.404 (0.095-1.710)  4.099 (1.535-10.946)  1.545 (0.464-5.148) | ***P***  ***0.05***  *0.11*  *0.25*  *0.10*  *0.22*  ***<0.01***  *0.48* |

^a^HR= Hazard Ratio; ^b^95%IC=95% Confidence Interval; ^c^RAB25 = RAB25 positive vs RAB25 negative; ^d^Sex: Female vs Male; ^e^Age: <60 years old vs >60 years old; ^f^Alcohol consumption= yes vs no; ^g^Tobacco consumption: yes vs no; ^h^N^-^ vs N^+^= node negative versus node positive; ^i^Tumor size= T1-T2vsT3-T4.
