# Supplementary material for: High RAB25 expression is associated with good clinical outcome in patients with locally advanced head and neck squamous cell carcinoma
Source: Cancer Med. 2013 Oct 31;2(6):950–63. doi: 10.1002/cam4.153 (PMC3892400; doi:10.1002/cam4.153)
Supplement: Supplementary file 6 [file cam40002-0950-SD6.doc]

Supplementary Table 2. Authentication of the HNSCC cell lines used in the study.

**a) STR profiles for the HNSCC cell lines analyzed in the study obtained using the Cell ID system**

| **CELL LINE** | **AMELOGENIN** | **CSF1PO** | **D13S317** | **D16S539** | **D5S818** | **D7S820** | **THO1** | **TPOX** | **vWA** | **D21S11** |
| --- | --- | --- | --- | --- | --- | --- | --- | --- | --- | --- |
| SCC-9 | X,Y | 11 | 9 | 10, 11 | 12 | 8 | 8,9 | 9,11 | 17 | ­­ND |
| SCC-25 | X | 10 | 13 | 11, 12 | 12 | 12 | 8 | 8,12 | 17,19 | ND |
| Fadu | ND | 12 | 8, 9 | 11 | 12 | 11, 12 | 8 | 11 | 15, 17* | 31.2 |
| UM-SCC-22A | X | 10 | 8, 12 | 9, 11 | 12, 12 | 8,9 | 6 | 8, 11 | 15, 18 | 28 |
| UM-SCC-22B | X | 10 | 8, 12 | 9, 11 | 12 | 8, 9 | 6 | 8, 11 | 15, 18 | 28 |
| UM-SCC-74B | X | 12 | 12 | 10, 12 | 12 | 11 | 6, 9.3 | 8 | 15, 16 | 30, 34.2 |
| K565 ^1^ | X | 9, 10 | 8 | 11, 12 | 11, 12 | 9, 11 | 9.3 | 8,9 | 16 | 29, 30, 31 |

^1^ Cell line used as an intrinsic control ND: Not detected *allelic loss

Cell line authentication was performed using the Cell ID system (Promega, USA) following manufacturer’s instructions. DNA was extracted from cells using the Wizard Genomic DNA purification Kit (Promega, USA) and quantified spectrophotometrically. For each cell line, 200 ng of DNA was amplified in a GeneAmp PCR 9700 system. STR markers were analyzed using a 3130xl Genetic Analyzer and GeneMapper v4.0 software.

**b) Reference STR profiles**

| **CELL LINE** | **AMELOGENIN** | **CSF1PO**^4^ | **D13S317** | **D16S539**^4^ | **D5S818** | **D7S820** | **THO1**^4^ | **TPOX**^4^ | **vWA** | **D21S11**^5^ |
| --- | --- | --- | --- | --- | --- | --- | --- | --- | --- | --- |
| SCC-9 ^1^ | X,Y | 11 | 9 | 10, 11 | 12 | 8 | 8,9 | 9,11 | 17 | ­­─ |
| SCC-25 ^1^ | X | 10 | 13 | 11, 12 | 12 | 12 | 8 | 8,12 | 17,19 | ─ |
| Fadu ^1^ | ND | 12 | 8, 9 | 11 | 12 | 11, 12 | 8 | 11 | 15, 17, 18 | ─ |
| UM-SCC-22A^2^ | X | ─ | 8, 12 | ─ | 12, 12 | 8,9 | ─ | ─ | 15, 18 | 28 |
| UM-SCC-22B^2^ | X | ─ | 8, 12 | ─ | 12 | 8, 9 | ─ | ─ | 15, 18 | 28 |
| UM-SCC-74B^2^ | X | ─ | 12 | ─ | 12 | 11 | ─ | ─ | 15, 16 | 30, 34.2 |
| K565 ^3^ | X | 9, 10 | 8 | 11, 12 | 11, 12 | 9, 11 | 9.3 | 8,9 | 16 | 29, 30, 31 |

^1^ Original STR profiles described in the ATCC Data Base ([http://www.lgcstandards-atcc.org](http://www.lgcstandards-atcc.org/))

^2^ Original STR profiles for the UM-SCC cell lines described by Brenner et al. ( Brenner, JC.Head and Neck 2010)

^3^ Original STR profile for the K565 cell line used as an intrinsic control

^4^ CSF1PO, D16S539, THO1 and TPOX STR loci were not included in the Profiler Plus PCR Amplification Kit (Applied Biosystems, Foster City, CA) used in the study described by Brenner et al.

^5^ D21S11 STR locus was not included in the ATCC Data Base.

ND: Not detected
